# The Role of Research in Viral Disease Eradication and Elimination Programs: Lessons for Malaria Eradication

**DOI:** 10.1371/journal.pmed.1000405

**Published:** 2011-01-25

**Authors:** Joel G. Breman, Ciro A. de Quadros, Walter R. Dowdle, William H. Foege, Donald A. Henderson, T. Jacob John, Myron M. Levine

**Affiliations:** 1 Fogarty International Center, National Institutes of Health, Bethesda, Maryland, United States of America; 2 Sabin Vaccine Institute, Washington (D.C.), United States of America; 3 Task Force for Global Health, Decatur, Georgia, United States of America; 4 The Bill & Melinda Gates Foundation, Seattle, Washington, United States of America; 5 Center for Biosecurity, University of Pittsburgh Medical Center, Baltimore, Maryland, United States of America; 6 Christian Medical College, Vellore, India [retired]; 7 Center for Vaccine Development, University of Maryland School of Medicine, Baltimore, Maryland, United States of America

## Abstract

By examining the role research has played in eradication or regional elimination initiatives for three viral diseases—smallpox, poliomyelitis, and measles—we derive nine cross-cutting lessons applicable to malaria eradication. In these initiatives, some types of research commenced as the programs began and proceeded in parallel. Basic laboratory, clinical, and field research all contributed notably to progress made in the viral programs. For each program, vaccine was the lynchpin intervention, but as the programs progressed, research was required to improve vaccine formulations, delivery methods, and immunization schedules. Surveillance was fundamental to all three programs, whilst polio eradication also required improved diagnostic methods to identify asymptomatic infections. Molecular characterization of pathogen isolates strengthened surveillance and allowed insights into the geographic source of infections and their spread. Anthropologic, sociologic, and behavioural research were needed to address cultural and religious beliefs to expand community acceptance. The last phases of elimination and eradication became increasingly difficult, as a nil incidence was approached. Any eradication initiative for malaria must incorporate flexible research agendas that can adapt to changing epidemiologic contingencies and allow planning for posteradication scenarios.

## Abstract 2

Using their experiences from, and analysis of, global campaigns to eradicate smallpox, poliomyelitis, and measles, Myron Levine and colleagues derive lessons for malaria eradication.

**Summary Points**

- Lessons from the smallpox, poliomyelitis, and measles eradication/elimination initiatives (in particular, the importance of starting laboratory, clinical, and field research early in the program and continuing research in parallel) should be incorporated into any malaria eradication initiative from the onset
- Vaccines are likely to be the lynchpin interventions of elimination/eradication programs, but ongoing research will be needed to improve formulations, delivery, and immunization schedules
- Surveillance will be critical throughout any elimination/eradication initiative, coupled with improved diagnostic methods to detect asymptomatic infections and low rates of transmission
- Because socio-cultural, religious, and local politics can impede eradication efforts, it is prudent to support research into improving ways to communicate effectively with local populations about the disease and the interventions to eradicate it
- A cross-cutting theme among the viral disease programs is that interrupting the last vestiges of transmission is particularly problematic and requires allocation of many resources including support for focused “last kilometre” research activities

## Introduction

Despite a previous global eradication campaign (1955–1969), malaria continues to be a major public health problem. Faced with hundreds of millions of malaria cases annually and nearly a million deaths, the international community is renewing efforts to eradicate this disease. But, initiatives for national or regional elimination or global eradication of any disease represent complex efforts that consume vast financial, health services, and infrastructural resources and require decades of commitment. Such programs demand sound scientific underpinnings and management structures that can adapt to changing epidemiologic scenes and can learn from the experiences of previous programs. Herein we describe three viral disease elimination/eradication efforts whose research agendas offer lessons for malaria scientists and public health program managers. The disease elimination programs we consider are smallpox (the one human infectious disease successfully eradicated), poliomyelitis (transmission of wild-type 2 poliovirus was interrupted globally since 1999, although transmission of types 1 and 3 continues in several countries), and measles (whose transmission has been eliminated in the Americas and in several countries worldwide). Each author has participated in one or more of these eradication/elimination initiatives and some also have experience in malaria research.

Throughout this article we use the following terms to denote progressive decreases in the extent of human disease and transmission of agent, as a result of deliberate interventions [1]. “Control” is the reduction of incidence of a disease to an arbitrary level whereupon it is no longer a public health priority. “Elimination” is the interruption of transmission of the pathogen when disease incidence becomes zero in a population within a large defined geographic area (e.g., one or more countries). A caveat in measles and polio elimination initiatives is that imported cases may appear in a country without indigenous transmission, i.e., a country that has achieved elimination. Elimination is considered to remain intact, so long as the importations are contained and do not ignite anew extended indigenous transmission. Finally, “eradication” signifies the interruption of transmission of a pathogen worldwide and a reduction in disease incidence to zero; this assumes that surveillance systems could detect transmission, if any. Theoretically, eradication should obviate the need for further control measures other than surveillance (as with smallpox).

Aside from the common requirements for adequate resource commitment, broad advocacy and political will relevant to all disease eradication initiatives, there are biologic and epidemiologic factors that specifically affect the feasibility of eradication of smallpox, polio, measles, and malaria. Table 1 summarizes these salient factors. Table 2 provides illustrative examples in which research played an important role in the eradication of smallpox, the near-eradication of polio, and the elimination of measles from the Americas and some other countries. From these experiences, lessons were learned that are applicable to the Malaria Eradication Program and that should, we believe, be incorporated in the Malaria Eradication Research Agenda (malERA) described in this Supplement.

**Table 1:** A comparison of the inherent salient features of smallpox, polio, measles, and malaria infections that favour or impede elimination of the disease and the most effective past and current interventions.

Feature	Smallpox	Polio	Measles	Malaria
**Disease syndrome is recognized by the public**	Yes	Yes (paralytic form)	Yes	Variable
**Extent of clinical expression**	100%	<1% (many subclinical and nonparalytic cases)	∼100%	Often low
**Specificity of the clinical disease**	High	High for paralytic disease; low for nonparalytic disease	Moderate	Often low
***n*** ** serotypes or species**	2: *V. major* (high case fatality) and *V. minor* (low case fatality)	3	1	5^a^
**Reservoir**	Humans	Humans	Humans	Humans (except for *P. knowlesi*)^a^
**Transmissibility**	Usually low to moderate	High	Very high	Variable
**Seasonality**	Yes (regional)	Yes (regional)	Yes (regional)	Often
**Incubation period (d)**	12–14	6–20	9–13	∼12
**Immunity follows a single clinical infection**	Yes	Yes (type specific)	Yes	No^b^
**Interventions**	Vaccine (live)	Vaccines (live oral and killed parenteral)	Vaccine (live subcutaneous)	ITNs; ACTs; IRS; IPTp; IPTi

a *P. falciparum*, *P. vivax*, *P. malariae*, and *P. ovale* are restricted to human hosts. *P. knowlesi*, which mainly infects nonhuman primates, can also cause disease in humans following natural transmission.

b However, the development of immunity against clinical disease follows repeated infections.

ACT, artemisinin combination therapy; IPTi, intermittent preventive treatment in infants; IPTp, intermittent preventive treatment in pregnancy; IRS, indoor residual spraying; ITN, insecticide treated bednets.

**Table 2:** Research outputs that contributed to the eradication of smallpox and the regional elimination of polio and measles (or outputs that are still undergoing evaluation or development): Lessons for the rejuvenated Malaria Eradication Program.

Research Output	Smallpox	Polio	Measles	Malaria
Basic research	Heat-stable vaccine; Bifurcated needle; Differentiation of orthopoxviruses based on genomic sequence analysis; Search for candidate antiviral drugs with activity against *Variola* (disappointing results in clinical trials)	Identification of 3 serotypes; Development of live and killed virus vaccines; Modern monovalent (type 1 or 3) and bivalent (types 1 and 3) vaccines; Sequencing of viral isolates; Search for safe and effective antiviral drugs	Live measles vaccine strains; IgM measles antibody diagnostics; Oral fluid-based diagnostic assays; Sequencing of viral isolates to obtain epidemiologic insights; Measles H DNA vaccine (to prime very young infants immunologically so they can respond safely and effectively to current live vaccine)	Biology of liver stage parasites; *In vitro* culture of *P. vivax*; Sensitive, simple, point of care diagnostics to detect both symptomatic and asymptomatic infections; Single encounter radical cure and prophylaxis drug; Vaccines to interrupt transmission; New effective insecticides that are safe for humans
Clinical research	Immunogenicity of vaccine administered by new methods of delivery (e.g., Ped-O-Jet; bifurcated needle); Evaluation of antiviral agents (marboran, cytosine, adenine arabinoside)	Immunogenicity of tOPV in different settings; Immunogenicity of monovalent and bivalent vaccines; Duration of OPV excretion by immunocompromised subjects	Identification of a correlate of protection (serum plaque reduction neutralizing antibody); Immune responses following initial immunization and following booster dose; Respiratory tract administration of vaccine by small particle aerosol or by large droplet spray	Improved measures of immune response; Identify immunologic correlates of protection
Field research	Definition of transmission indices; Surveillance/containment strategy; Discovery of monkeypox	Impact of national and subnational mass immunizations; Identification of outbreaks due to circulating vaccine-derived polioviruses; Anthropological and sociological studies to enhance local support for vaccination	Identification of the “window of vulnerability” in infants; Impact of national and subnational mass immunizations; Coupling mass measles immunization with OPV and antihelminthic administration and bednet distribution	Improved methods to measure malaria transmission in different settings; Improved methods for measurement of malaria morbidity and mortality; Studies of local vectors to identify points of intervention

## Lesson 1. Research Should Accompany Elimination/Eradication Efforts from the Outset

The foremost lesson learned from eradication/elimination efforts for viral diseases is that a flexible research agenda must be initiated early, prior to or concomitant with the launch of eradication interventions.

### Smallpox

Since 1959, when the World Health Assembly (WHA) resolved to undertake global smallpox eradication, research played an integral role in every facet of its implementation [2]. Without the products of field and laboratory research and their incorporation into the program, eradication would not have been achieved. Research improved vaccine production methods to assure the universal availability of potent, heat-stable products [3],[4] and provided improved instruments and methods for performing vaccination [5],[6]. Field studies yielded new insights into the epidemiologic behaviour of smallpox under differing circumstances and identified optimal preventive and containment methods for control, elimination, and eradication [2],[7]–[10].

Between 1959 and 1966, progress in smallpox eradication was limited. Then, in 1966 the WHA intensified the effort by allocating US$2.4 million for the program. An overall strategy was formulated that included vaccination of 80% of the population in each country using vaccines of assured potency and establishment in all countries of a weekly reporting system from all health units with plans to vaccinate contacts and neighbours of all cases to stop each outbreak rapidly—an approach termed “surveillance-containment” [11]. In 1967, 43 countries reported 132,000 cases of smallpox, but studies revealed that only 1%–2% of cases were being reported at that time. [12]. The goal was to stop smallpox transmission in 10 years. The last case occurred 10 years, 11 months, and 26 days later.

### Polio

Poliomyelitis (polio) was one of six childhood diseases targeted for control in 1974 by the World Health Organization (WHO) through the Expanded Program on Immunization (EPI). Research during the Smallpox Eradication Program confirmed the feasibility of coadministering multiple antigens and experience was acquired in managerial aspects of vaccine delivery and disease surveillance [13]. However, polio outbreaks continued in low- and middle-income countries, mostly tropical/subtropical, despite routine administration of trivalent oral polio vaccine (tOPV) [14].

In 1980, Brazil began coordinated mass administration of tOPV (supplementary immunization activity) twice annually to all children <5 years of age [15], and a dramatic reduction of paralytic polio incidence ensued. Encouraged by the success of this strategy, in 1985 the Pan American Health Organization resolved to eliminate polio in the Americas by 1990. In 1988, the WHA resolved to eradicate polio worldwide by 2000 [16] and the Global Polio Eradication Initiative (GPEI) was established in partnership with UNICEF, the US Centers for Disease Control and Prevention, and Rotary International; WHO was responsible for overall management, program implementation, and fundraising.

In GPEI's early years, with funding shortages and success of the program in the Americas, research was not a priority. Nevertheless, limited applied research, driven by emerging operational needs and gaps, led to advances in vaccine logistics, cold chains, monitoring and evaluations, laboratory methodology, and surveillance to detect cases of acute flaccid paralysis (AFP) [17].

Appreciation by GPEI of the need for intensified research grew as new programmatic challenges and findings about polio virology and epidemiology were encountered and posteradication questions emerged. With awareness that additional approaches would be essential if the target date for global interruption of transmission was to be met, a Global Technical Consultative Group was convened in 1996 to address challenges in eradication progress [18]. Although polio due to type 2 wild poliovirus was eradicated globally in 1999, cases and outbreaks due to types 1 and 3 continued. And, rather than marking global eradication, the year 2000 saw an unexpected outbreak of 21 polio cases in Hispaniola caused by circulating vaccine-derived poliovirus [19]. Recognition that vaccine-derived poliovirus could cause epidemics of AFP reinforced the need for flexible research efforts to respond expeditiously to emerging needs [19],[20].

By 2004 [21], newer challenges to eradication in endemic regions were recognized, including low tOPV efficacy in certain populations, low herd immunity, and the participation of vaccinated children in wild poliovirus circulation—collectively considered as “failure-of-vaccine.” More frequent supplementary immunization activity proved to be inadequate, highlighting the need for research to elucidate virus transmission better and to identify correlates of protection relevant at the population level. Consequently, the WHO Polio Research Committee was established in 2008 to provide a forum for addressing timely research questions [22]; the Advisory Committee on Poliomyelitis Eradication now provides oversight for research and application of findings in program implementation [23].

In retrospect, anticipating research questions was difficult when the path to polio eradication seemed straightforward. Today, 10 years past the original eradication goal, research has greatly expanded, including ongoing research in operations, evidence-based communication strategies to overcome socio-cultural or religious belief-based resistance to vaccination [24], vaccines and immunity, molecular epidemiology, mathematical modeling [25], and a search for antivirals to curtail virus shedding.

The lesson learned from GPEI that research should accompany elimination/eradication efforts from the outset applies directly to the unsuccessful Malaria Eradication Program of 1955–1969. This Program relied heavily on indoor spraying with residual insecticides and detection of cases and treatment with chloroquine as the primary interventions. Without a strong ongoing research program within a flexible infrastructure, this program could not respond adequately to the emergence of widespread mosquito resistance to DDT and parasite resistance to chloroquine.

### Measles

Measles, one of the most communicable of all infectious diseases, exhibits an extraordinary propensity to reach susceptible individuals even when they constitute only a small proportion of the population [26]. In the prevaccine era, all children experienced measles unless they lived in remote areas. [27]. The clinical expression of infection approached 100% and led to life-long protection against disease; the occasional individuals with subclinical infection did not, apparently, transmit virus.

The case fatality rate of measles in malnourished infants in developing countries exceeds 20% [26]. In 1999, measles was the third most common cause of death among children <5 years of age in developing countries and the most common vaccine-preventable cause. The gravity of measles disease and its complications and the magnitude of the human and economic tolls exacted are often insufficiently perceived by health professionals and the public: even in industrialized countries measles can be severe with at least one case among every 1,000 proving fatal [28].

In 1994, health ministers in the Americas committed to eliminating measles from the Western Hemisphere [29], using a triple pincer vaccination strategy consisting of a one-time “catch-up” campaign targeting children 9 months through 14 years of age (to interrupt wild-virus circulation), strengthened services to “keep-up” routine measles vaccination in infants, and “follow-up” campaigns to maintain immunity in the preschool age group. Indigenous transmission was interrupted by 2003, despite repeated importations of measles from Europe and Japan [29].

Since 2000, considerable progress has been made worldwide in diminishing mortality from measles through immunization campaigns, particularly in sub-Saharan Africa [30]. When high coverage is achieved, campaigns eliminate measles virus from the community and indirectly protect young infants by diminishing the force of infection. During field research in Togo in 2004 [31], a national campaign to administer measles vaccine to all children 9–59 months of age was coupled with giving oral polio vaccine (OPV), an oral antihelminthic, and insecticide-treated bednets; >90% vaccination coverage was achieved. However, in some African countries repetitive mass campaigns are proving difficult to sustain and measles mortality in young children remains problematic [32].

Research that helped interrupt transmission of measles in the Western Hemisphere and to diminish measles mortality in Africa includes studies of measles transmission in different populations, improved diagnostic tests and sero-epidemiologic methods, molecular finger printing to determine the geographic origin and relatedness of measles virus isolates [33],[34], and improved methods of immunizing against measles using existing vaccines [35]. Other research focuses on developing new vaccines to immunize high-risk target groups (e.g., very young infants) who cannot be effectively immunized with currently licensed measles vaccines [36].

## Lesson 2. The Reservoirs of Infection and Degree and Specificity of Clinical Expression Influence the Eradication Program

A feature common to smallpox, poliomyelitis, measles, and *Plasmodium falciparum* and *Plasmodium vivax* malaria is that humans constitute the sole reservoir of these pathogens; one need not worry about animal or environmental reservoirs as sources of reintroduction into human populations.

### Smallpox

The discovery of human monkeypox exemplifies the importance of research to confirm the absence of a nonhuman reservoir for diseases targeted for eradication, including malaria. The monkeypox exanthem in humans resembles smallpox, albeit with milder clinical symptoms and lower fatality rates. Accordingly, during and after smallpox eradication in Africa there was concern about the possible persistence of this orthopoxvirus virus in natural settings [37]. Epidemiologic and laboratory research on monkeypox in enzootic areas of Africa [38]–[40] confirmed that it did not spread easily in human populations and posed only a small threat for becoming an endemic human illness [39],[40], even though some localized foci have been identified [37]. Recent reports of human infections with the nonhuman primate parasite *Plasmodium knowlesi* [41], raise concerns that, in certain ecologies, *P. knowlesi* may increase in humans as *P. falciparum* disappears.

### Polio

Smallpox and measles have ∼100% clinical expression in immunocompetent persons and asymptomatic chronic infections do not occur. By contrast, many asymptomatic or mild cases of poliovirus, *P. falciparum*, and *P. vivax* infection occur for every clinical case. Early epidemiologic field research of polio identified ∼150 infections that did not progress to paralysis for each case of AFP [42]. Moreover, persons with B-cell immunodeficiencies can chronically excrete vaccine polioviruses. These hidden reservoirs make polio and malaria eradication fundamentally more difficult than smallpox. Improved diagnostic tests are needed to identify persons infected with polio and malaria, as cases become less common.

### Measles

Akin to the clinical confusion of measles with rubella and other febrile exanthemata, clinical *P. falciparum* and *P. vivax* infections are easily confused with many other febrile disorders. In another parallel, immunocompromised individuals with measles giant cell pneumonia may shed virus without having a rash and malaria-immune individuals may have parasites in their blood in the absence of clinical symptoms and may act as infectious source for the mosquito vector. The most vexing issue in malaria elimination/eradication is *P. vivax* hypnozoites, a form of the parasite resident in the liver that creates persistent (for years), silent infection that is nonresponsive to standard treatment for clinical malaria. The only current drug effective against *P. vivax* hypnozoites is the 8-aminoquinoline primaquine.

To summarize, the lesson learned here is that malaria eradication will be facilitated by improved diagnostics that can detect mild and asymptomatic blood infections and that can identify asymptomatic persons harboring *P. vivax* hypnozoites. A corollary lesson is that high priority should be placed on developing new, well-tolerated drugs to treat persons with latent *P. vivax* infection, particularly individuals genetically deficient in glucose 6-phosphate 1-dehydrogenase (who develop hemolytic anemia when treated with primaquine). malERA's concept of developing a Single Encounter Radical Cure and Prophylaxis (“SERCaP”*)* drug, if successful, would accomplish that.

## Lesson 3. The Critical Role of Surveillance

A theme common to the smallpox, polio, and measles eradication/elimination programs is the critical role that surveillance has played in every phase, including quantification of the burden at the onset of the program; monitoring progress of the program at local, national, and global levels; intensive searches for the last cases and infected persons; and documentation of the interruption of transmission. The critical role of surveillance necessitated research to develop new epidemiologic surveillance systems for all three diseases and, for measles and polio, sero-epidemiologic methods, tests to identify asymptomatic carriers, and molecular methods to establish the geographic source and relatedness of isolates from outbreaks and clusters over different time periods. This lesson is directly applicable to the Malaria Eradication Program, which will need to assure that adequate surveillance methods and techniques are in place to monitor the effectiveness of the program.

### Smallpox

The magnitude of the smallpox problem was largely unknown in 1959, despite the International Health Regulation that all smallpox cases be reported. Finding and controlling outbreaks quickly was essential for the containment strategy. Accordingly, within each country, all health care facilities were asked to provide a weekly report about smallpox cases. Every 3 weeks, international surveillance reports were prepared and widely distributed that charted progress by country, informed new findings through research, and recommended changes in strategy. These reports and special national reports developed by some countries were invaluable in rapidly informing all concerned about progress in the program and in conveying new discoveries and new directions for the program.

Another aspect of smallpox eradication that might be relevant to the malaria elimination/eradication program is the rigorous program of certification of absence of smallpox that began in the 1970s and that was intensified until the WHA confirmed global eradication in 1980. Tens of thousands of specimens from persons with “fever and rash” were collected with well-publicized rewards being offered to persons reporting any patient with confirmed smallpox.

### Polio

Pathogens other than polioviruses also cause AFP. A measure of the quality of polio surveillance is the adequacy of detection of AFP cases and the proportion of cases from whom stool specimens are obtained for virological analysis. Moreover, paralytic polio cases represent only the tip of the epidemiologic iceberg. Thus, polio shares with malaria the attribute that many persons harbouring infection will be clinically unsuspected. In the context of eradication, all infected individuals are epidemiologically important [43]. Consequently, malERA has rightly given high priority to the development of improved tests to detect clinically typical, mild and asymptomatic *Plasmodium* infections and to assess the extent of transmission.

### Measles

Measles outbreaks must be detected and curtailed to limit transmission following importations. For outbreak detection, specific, practical, and rapid measles diagnostic tests are needed. Research developed such tests and the strategies to use them. Serum specimens and either urine or nasopharyngeal samples are obtained from suspect measles cases and, as appropriate, from contacts [44]. The serum is tested for measles-specific immunoglobulin M (IgM) antibodies indicative of acute infection. A noninvasive alternative involves collecting oral fluid [45]. Measles virus in urine or nasopharyngeal specimens is detected by culture or reverse transcriptase PCR. Unfortunately, these tests are not suitable for point-of-care diagnosis. A simple, rapid, inexpensive, sensitive, and specific point-of-care diagnostic for measles will facilitate eradication efforts. Similarly, malERA has identified the need for a sensitive, specific, and inexpensive diagnostic test amenable to use in the field.

## Lesson 4. Molecular Epidemiology

Research fostered by the viral disease eradication/elimination programs has shown how molecular tools add precision to surveillance. The molecular epidemiologic evaluation of plasmodial parasites will be similarly helpful, particularly in the later stages of a Malaria Eradication Program; research in this area should be encouraged.

### Smallpox

Genetic analysis of isolates of orthopoxviruses from patients and animals has shown the important differences among smallpox viruses (*Variola major* and *V. minor*), monkeypox, and vaccinia that are useful for surveillance [46],[47].

### Polio

Partial genomic sequencing of all wild poliovirus isolates is undertaken to determine genetic relatedness. Each 1% difference between two isolates correlates with approximately 1 year of undetected circulation between the specific chains of transmission. A difference of >1.5% suggests undetected past transmission, thereby identifying inefficient surveillance systems. In addition, timely genome sequencing and construction of phylogenic trees make it possible to assess eradication progress through genetic cluster elimination, to identify human reservoirs, to differentiate indigenous from imported viruses, to identify surveillance gaps (through isolates without recent parental strains), and to identify vaccine-derived polioviruses and quantify their period of circulation [20],[48].

### Measles

Genotyping of measles viruses allows identification of the geographic origin of imported viruses/cases and provides a means of tracking epidemiologic relationships among cases in the same or separate outbreaks [33],[34].

## Lesson 5. The Pivotal Role of Vaccines as a Tool for Disease Eradication

The eradication of smallpox and of type 2 poliovirus infection globally, and the elimination of polio and measles from various regions and countries was achieved using vaccines as the primary intervention tool. As malaria transmission diminishes, other interventions (e.g., vector control, insecticide-impregnated bednets, new drugs, etc.) will surely play critical roles, but the lesson from the viral disease programs is that vaccines that interrupt transmission could play a critical role in helping to eradicate malaria.

### Smallpox

Until the 1960s, smallpox vaccine was typically a liquid product of suboptimal potency, readily inactivated by heat within a few days. Industrial process research developed a method for producing heat-stable, freeze-dried smallpox vaccine [4] that could withstand temperatures of 37°C for at least 1 month. With technical assistance from industrialized countries, >80% of lyophilized smallpox vaccine of acceptable quality was being manufactured in developing countries within 6 years of the eradication program starting. Having smallpox vaccine that did not require refrigeration was of immeasurable practical importance in the field [11].

At the onset of the eradication program, age-old, traditional techniques of scratching or pressing the vaccine into the skin frequently failed to immunize. New vaccination techniques were introduced that permitted more rapid and effective inoculations. Jet injectors were perfected and field tested that could vaccinate hundreds of people per hour. By 1971, the injectors were superseded by a simple two-pronged (bifurcated) needle [49]. WHO tested these needles for a unique multiple-puncture vaccination technique. Successful vaccination responses approached 100%, less vaccine was required for each vaccination, instruction in vaccination required only ∼15 minutes, and the needles could be sterilized and reused repeatedly. In Africa and Asia, with a good working rapport with villagers and their leaders, a vaccinator with bifurcated needles could average 500 vaccinations per day. To measure vaccination coverage and vaccine “take” rates (vesicle or early crusting lesion on the skin 1 week after vaccination), a sample survey of villagers was routinely checked [50].

The impact of the bifurcated needle in improving the logistics of smallpox vaccination was immense. A possible analogous situation for malaria eradication may arise with the need to identify practical ways to deliver the promising attenuated sporozoite vaccines that are under development [51]–[53].

Smallpox field research may also provide lessons for malaria eradication efforts. For example, smallpox outbreak containment teams that were deployed to the field to determine how smallpox outbreaks spread and to vaccinate contacts and neighbours of patients discovered that smallpox did not spread as rapidly and widely as textbooks described. Chains of smallpox transmission could be broken in most areas by the surveillance-containment approach, and this approach was soon given priority over mass vaccination. Similarly, field research showed that smallpox vaccine protection lasted at least 10 years, not 3–5 years as traditionally thought. Recent research on immunologic memory has established the basis for the long-lived protection [54].

### Polio

Research in the 1950s created two polio vaccines—an oral approach based on three live attenuated poliovirus strains (originally administered sequentially but subsequently licensed as a trivalent formulation) [43], and an intramuscular vaccine consisting of three formalin-inactivated polioviruses. Although both vaccines drastically diminished polio cases in industrialized countries, tOPV was selected as the lynchpin of the GPEI, being less expensive and easier to administer. Failure to achieve the goal of polio eradication by 2000 was attributed to inadequate vaccination coverage and research recommendations were primarily operational in nature. However, it has since become apparent that there are major gaps in our understanding of immune mechanisms. Current research priorities include the development of surrogate measures of mucosal immunity and interventions to boost and prolong immunity, and the determination of the relationship between waning immunity and virus circulation. Research is also addressing the observation that tOPV in infants appears to be less immunogenic in some areas in India than elsewhere [55]–[58].

Recognizing that type 2 polio has been eradicated since 1999 but that type 1 and 3 disease continues, an accelerated collaborative research and development effort undertaken with industry resulted in the licensure and use of monovalent type 1 and 3 vaccines and a novel bivalent (types 1 and 3) OPV formulation [23],[59]. Deleting the more immunogenic and dominant type 2 virus that interferes with responses to the type 1 and 3 viruses allows enhanced serological responses to types 1 and 3. The bivalent vaccine has now become the preferred tool in supplemental immunization campaigns.

### Measles

Cell culture propagation of measles virus in 1954 was followed by development of the first generation of parenteral live measles vaccines, which were protective but associated with unacceptably high rates of febrile reactions. Further research yielded the current well-tolerated vaccines. Inactivated measles-virus vaccines had also been licensed in 1963 based on safety, immunogenicity, and short-term efficacy data [60]. However, immunity was short-lived; postlicensure surveillance revealed that some vaccine recipients developed a syndrome of atypical measles when subsequently exposed to wild measles virus [61],[62]. Accordingly, inactivated measles vaccine use was discontinued by 1967.

The fall in measles cases following introduction of the first generation measles vaccine in the United States in 1963 prompted epidemiologists to predict that measles could be eliminated country-wide by 1967, if vaccine could be administered routinely to infants and to susceptibles at school entry and if surveillance and epidemic control could be strengthened [63]. Although measles incidence fell by >90% by 1967, it took 26 more years until indigenous transmission was interrupted in the United States. This achievement required a routine second dose of vaccine before school entry and a reduction in imported infections consequent to enhanced measles control elsewhere [64].

By 1999, most measles deaths were occurring among children in the Indian sub-continent and sub-Saharan Africa, despite recommendations that measles vaccine should be given routinely to infants ∼9 months of age. A notable proportion of these measles deaths clustered among young infants during their “window of vulnerability” (approximately 4–9 months of age) [65], when falling titres of maternally derived measles antibodies no longer protect against disease but nevertheless interfere with successful immunization. Reports that immunogenicity could be enhanced in infants <6 months of age by administering high-titre vaccine generated optimism that a solution to protecting young infants might be at hand [66]. However, this approach was soon abandoned when long-term follow-up revealed unexplained increased mortality in female children [67].

Three new research efforts are addressing ways to protect young infants in developing countries, to provide adjunct tools for measles elimination [68]. The first involves repetitive follow-up mass immunization of children with the existing vaccines to indirectly protect young infants [30]. The second involves clinical trials to allow licensed vaccine to be administered to the respiratory tract by small particle aerosol, thereby making mass immunization simpler and safer [35]; clinical research has shown that vaccine delivered to the nasal mucosa by large droplet spray is ineffective [69]. The third research effort has resulted in development of a candidate measles DNA vaccine encoding the hemagglutinin (H) antigen of measles virus [36].

## Lesson 6. Modes of Transmission and Modeling

Smallpox and measles viruses are transmitted by the respiratory route (droplets/aerosol), while polio is mainly transmitted by the fecal-oral route in developing countries. Although modeling played no role in smallpox eradication, it has been extremely useful in the GPEI as a valuable epidemiologic research tool, for addressing economic issues, and for providing insight into future programmatic options [25]. Modeling research is currently addressing the risks of virulent vaccine-derived poliovirus that may be chronically shed by immunodeficient individuals and from circulating vaccine-derived poliovirus, after OPV is withdrawn posteradication [70]. Similarly, measles was one of the first infectious diseases studied with models, and models are now being used to elucidate better the epidemiologic behaviour of measles and predict the effect of interventions [71],[72]. Although the ability to generalize from models is debated [73], there is consensus that the quality of input data is steadily improving, even as the epidemiology of measles is changing globally.

Malaria, spread by female *Anopheles* mosquitoes, has a more complex transmission than these viral infections, which allows transmission to be decreased by targeting to control the vector or vector-host contact, as well as by changing susceptibility of the human host. Modeling is therefore particularly important to predict the effect of various interventions used independently and in unison on the transmission of malaria. It can also identify ways to minimize and delay parasite resistance to drugs [74] and should be an integral part of any malaria elimination/eradication program, as recognized by malERA.

## Lesson 7. Sociological, Anthropological, Cultural, and Religious Issues

Another lesson for malaria from the viral eradication/elimination programs is the important role that socio-cultural, religious, and local political factors play in public perception of the disease and of the main intervention tools of the eradication program; these factors can accelerate or impede eradication efforts. It is prudent to support research on these issues and on improving ways to communicate effectively with local populations. In this area of research, one size does not fit all.

### Smallpox

Smallpox was a severe, commonly lethal infection that often left survivors scarred and occasionally blind. Thus, in most endemic areas smallpox was recognized and feared by the population. Aversion to vaccination was not, therefore, a major impediment during the Smallpox Eradication Program.

### Polio

As paralytic polio (a relatively rare disease) diminished in incidence and became less of a threat, it became increasingly difficult to motivate populations to continue support for eradication activities. The GPEI and public health authorities worldwide became concerned by events in Nigeria in 2003–2004 that set polio eradication back there and in much of Africa. In late 2003, several states in northern Nigeria refused to participate in national mass immunization campaigns. Religious and political leaders in three states counseled parents against having their children immunized, preaching that the vaccine was contaminated with antifertility hormones, HIV, and cancer-inducing agents [75]. Only after a Nigerian team (including members from the affected states) visited a manufacturer of OPV in Indonesia, a Muslim country, did the state governments accept that OPV was safe [75]. Confidence was restored and progress in polio eradication has since been achieved in Nigeria and elsewhere in Africa [76]. The lesson here is that evidence-based communication strategies must be carefully planned and implemented to overcome resistance to vaccination that originates from socio-cultural or religious beliefs [24],[77].

### Measles

A potential barrier to global eradication of measles is the poor measles vaccine coverage in many industrialized countries (in Europe and Japan) where strong antivaccine movements specifically target the measles vaccine. Without supporting scientific evidence, these antivaccine groups indict measles vaccine as a cause of autism and other chronic disorders. Continuing measles transmission in such industrialized countries maintains a reservoir that imperils elimination efforts in other countries. Further research in communications, anthropology, and sociology must be undertaken to find ways to counteract the antivaccine propaganda and increase the acceptance of measles vaccine.

## Lesson 8. The Concept of “The Last Kilometre”

A cross-cutting theme among the smallpox, polio, and measles eradication/elimination programs is that interruption of the last vestiges of transmission in a country or region is problematic and requires the allocation of as many resources as the early stages that achieved a 90%–99% reduction in incidence. Therefore, interventions often need to be modified, sometimes drastically, to complete the job of elimination.

Similarly, in the future, the final stages of the Malaria Eradication Program will likely confront barriers as complex, demanding, and refractory as ones encountered early in the program. Some will be resolvable only through directed, focused research. Thus, the rejuvenated Malaria Eradication Program should support a flexible research infrastructure that can adapt to the challenges.

## Lesson 9. Posteradication Agendas

The final lesson learned from the viral disease eradication programs is that discussion of posteradication scenarios, problems, and potential solutions must begin at the onset of the programs. Focused research can find early solutions for some posteradication issues. In the case of smallpox**,** affirmation of the eradication of smallpox was followed by a discontinuation of routine vaccination globally. The only way that smallpox disease can occur anew is if nefarious individuals with access to virus undertake a deliberate bioterror release. In the case of polio, however, since 2005, GPEI has been grappling with posteradication questions of use of OPV, the quandary of vaccine-derived poliovirus persistence, laboratory destruction and containment of poliovirus stocks, surveillance needs, vaccine compositions, and response strategies. These questions have become the drivers of a research agenda [78]. For measles, the major posteradication dilemma will be whether to continue routine immunization with the live measles vaccine. Given that in some industrialized countries, certain groups in the population view measles vaccine with more suspicion than the wild virus, it might be necessary to develop and utilize an alternative nonliving type of measles vaccine [36].

## Concluding Comments

Nine cross cutting lessons have been provided by these three vaccine-dependent eradication and elimination programs of viral diseases in which research was integral to guide program activities. These lessons will be useful to the revitalized Malaria Eradication Initiative. Research played a critical role in the Smallpox Eradication Program and is still contributing critically to the GPEI and measles elimination and mortality control programs. Despite having tools for primary prevention, considerable research has been essential to address geographic variations in the force of transmission of smallpox, polio, and measles and to adjust the tactical use of the preventive tools.

The ecology and epidemiology of malaria are far more complex than the ecology and epidemiology of these viral infections. Thus, if a global Malaria Eradication Initiative is revived, from the outset the Malaria Eradication Research Agenda should be incorporated as an essential component. Malaria eradication proponents should understand the importance of combining operational and research issues. Over time in successful elimination initiatives, the best researchers will see their ideas implemented and the best implementers will continue to ask what research could further improve operations.

## Abbreviations

AFP: acute flaccid paralysis
GPEI: Global Polio Eradication Initiative
OPV: oral polio vaccine
tOPV: trivalent oral polio vaccine
WHA: World Health Assembly
WHO: World Health Organization
